# Characterizing heart failure with preserved and reduced ejection fraction: An imaging and plasma biomarker approach

**DOI:** 10.1371/journal.pone.0232280

**Published:** 2020-04-29

**Authors:** Prathap Kanagala, Jayanth R. Arnold, Anvesha Singh, Daniel C. S. Chan, Adrian S. H. Cheng, Jamal N. Khan, Gaurav S. Gulsin, Jing Yang, Lei Zhao, Pankaj Gupta, Iain B. Squire, Leong L. Ng, Gerry P. McCann

**Affiliations:** 1 Aintree University Hospital and Clinical Research Fellow, National Institute for Health Research (NIHR) Leicester Biomedical Research Centre, Leicester, England, United Kingdom; 2 National Institute for Health Research (NIHR) Leicester Biomedical Research Centre, Leicester, England, United Kingdom; 3 Kettering General Hospital and National Institute for Health Research (NIHR) Leicester Biomedical Research Centre, Leicester, England, United Kingdom; 4 Bristol-Myers Squibb, Princeton, New Jersey, United States of America; Scuola Superiore Sant'Anna, ITALY

## Abstract

**Introduction:**

The pathophysiology of heart failure with preserved ejection fraction (HFpEF) remains incompletely defined. We aimed to characterize HFpEF compared to heart failure with reduced ejection fraction (HFrEF) and asymptomatic hypertensive or non-hypertensive controls.

**Materials and methods:**

Prospective, observational study of 234 subjects (HFpEF n = 140; HFrEF n = 46, controls n = 48, age 73±8, males 49%) who underwent echocardiography, cardiovascular magnetic resonance imaging (CMR), plasma biomarker analysis (panel of 22) and 6-minute walk testing (6MWT). The primary end-point was the composite of all-cause mortality and/or HF hospitalization.

**Results:**

Compared to controls both HF groups had lower exercise capacity, lower left ventricular (LV) EF, higher LV filling pressures (E/E’, B-type natriuretic peptide [BNP], left atrial [LA] volumes), more right ventricular (RV) systolic dysfunction, more focal and diffuse fibrosis and higher levels of all plasma markers. LV remodeling (mass/volume) was different between HFpEF (concentric, 0.68±0.16) and HFrEF (eccentric, 0.47±0.15); p<0.0001. Compared to controls, HFpEF was characterized by (mild) reductions in LVEF, more myocardial fibrosis, LA remodeling/dysfunction and RV dysfunction. HFrEF patients had lower LVEF, increased LV volumes, greater burden of focal and diffuse fibrosis, more RV remodeling, lower LAEF and higher LA volumes compared to HFpEF. Inflammatory/fibrotic/renal dysfunction plasma markers were similarly elevated in both HF groups but markers of cardiomyocyte stretch/damage (BNP, pro-BNP, N-terminal pro-atrial natriuretic peptide and troponin-I) were higher in HFrEF compared to HFpEF; p<0.0001. Focal fibrosis was associated with galectin3, GDF-15, MMP-3, MMP-7, MMP-8, BNP, pro-BNP and NTproANP; p<0.05. Diffuse fibrosis was associated with GDF-15, Tenascin-C, MMP-2, MMP-3, MMP-7, BNP, proBNP and NTproANP; p<0.05. Composite event rates (median 1446 days follow-up) did not differ between HFpEF and HFrEF (Log-Rank p = 0.784).

**Conclusions:**

HFpEF is a distinct pathophysiological entity compared to age- and sex-matched HFrEF and controls. HFpEF and HFrEF are associated with similar adverse outcomes. Inflammation is common in both HF phenotypes but cardiomyocyte stretch/stress is greater in HFrEF.

## Introduction

Heart failure with preserved ejection fraction (HFpEF) represents a growing yet incompletely understood clinical entity[1]. While heart failure with reduced ejection fraction (HFrEF) has been extensively studied, with a compelling evidence base, similar data are lacking in HFpEF[1]. Furthermore, whether HFpEF and HFrEF are part of the same syndrome or separate entities, remains subject to debate[2]. Most epidemiological and clinical trial data on HFpEF are based on imaging with echocardiography[3]. Cardiovascular magnetic resonance imaging (CMR) is the recognized gold standard for the majority of imaging parameters that comprise latest guidance on HFpEF, as well as for right ventricular (RV) assessment[1]. Furthermore, CMR provides unique tissue characterization properties for assessment of the extra-cellular space, namely late gadolinium enhancement imaging (LGE) for focal fibrosis[1, 4] and pre and post-contrast T1 mapping for extracellular volume (ECV)[5] quantification, a surrogate of interstitial fibrosis, both of which are implicated in HFpEF pathophysiology[2].

However, in-depth phenotyping of such HF groups with integrated CMR and extensive plasma biomarker profiling has yet to be performed. In this prospective, observational study, we compared CMR, echocardiography and circulating biomarkers between HFpEF, HFrEF and controls with the aim of gaining pathophysiological insights into the HFpEF syndrome. We further assessed whether clinical outcomes differed across the three groups.

## Materials and methods

### Study population

All subjects were recruited at a single tertiary cardiac centre as part of a prospective, observational, cohort study. The inclusion criteria for the HF groups were clinical[6] or radiographic evidence of HF and left ventricular EF on transthoracic echocardiography (TTE) of ≥50% for HFpEF or <40% for HFrEF. The exclusion criteria were: documented myocardial infarction (MI) in the preceding 6 months, suspected or confirmed cardiomyopathy (e.g. HCM, amyloid) or constrictive pericarditis, severe native valve disease, non-cardiovascular life expectancy <6 months, severe pulmonary disease (forced expiratory volume [FEV_1_] <30% predicted or forced vital capacity [FVC] <50% predicted), estimated glomerular filtration rate (eGFR) <30 ml/min/m^2^ and standard contraindications to CMR[4, 7]. At the time of study conception and conduct, owing to conjecture and lack of international consensus[2] regarding the existence of and clear diagnostic thresholds for HFpEF, patients with with mid-range EF (HFmrEF) were excluded to avoid uncertainty in the characterisation of HFpEF. The presence of diastolic dysfunction was not chosen as an inclusion criterion for HFpEF diagnosis since contemporary clinical trial data has also highlighted its presence at rest in only two thirds of HFpEF patients[8, 9]. Furthermore, elevated natriuretic peptides were also not a requirement for HFpEF diagnosis in our study since normal levels of natriuretic peptides have previously been observed in a significant proportion of such patients[8, 10].

Since the primary focus of our study was phenotyping HFpEF, we enrolled consecutively approached patients. HFrEF and control subjects were then recruited towards the end of the study. While it is well recognised from epidemiological data[3, 8, 11] that HFpEF patients are older and more frequently female, age- and sex-matching was performed in the HFrEF and control groups in order to limit the number of confounding variables.

Asymptomatic controls without known cardiac disease were recruited. Controls were recruited through advertising and none had been referred for a clinical CMR scan. Fourteen volunteers had also served as healthy controls in another study at our centre[12]. We did not exclude hypertensive controls (n = 19) since hypertension is highly prevalent in the general population without heart failure and is strongly associated with incident HFpEF[13] and we wanted to account for this potential confounder.

All subjects underwent comprehensive clinical assessment, blood sampling, TTE and CMR, completed the Minnesota Living with Heart Failure (MLHF) questionnaire[14] and six-minute walk test (6MWT)[15]. At baseline, coronary artery disease (CAD) was defined as either patient self-reporting of anginal symptoms, documented MI, coronary angiographic vessel luminal stenosis of ≥70% and/or coronary revascularisation based upon patients’ medical records. The results from the latest (last) chest radiographic reports prior to patients’ study visit were sourced from the electronic Hospital Radiology reporting systems. The study was approved by the United Kingdom National Research Ethics Service (reference: 12/EM/0222). Informed consent was provided by all subjects prior to participation. The study was conducted according to the Declaration of Helsinki and was registered with ClinicalTrials.gov (NCT03050593).

### Plasma biomarker assessment

At recruitment, blood was sampled for B-type natriuretic peptide ([BNP]—immunoassay, Siemens, Erlangen, Germany), haematocrit, haemoglobin, troponin-I (normal ≤40 ng/L, ultrasensitive, sandwich chemiluminescence assay, ADVIA 2400 analyser, Siemens, Erlangen, Germany) and renal function. Residual supernatant plasma was stored at -80°C in cryotubes prior to batch analysis at a later stage using a Luminex® bead-based multiplex assay[16], enabling high-throughput biomarker profiling as previously described[17].

The following plasma biomarkers implicated in HF and also recently evaluated using the same assay in the TOPCAT HFpEF clinical trial[18, 19] were profiled: suppression of tumorigencity-2 (ST2), galectin3, growth differentiation factor-15 (GDF-15), Tenascin-C, tissue inhibitor of metalloproteinases (TIMP-1, TIMP-4), matrix metalloproteinases **(**MMP-2, MMP-3, MMP-7, MMP-8, MMP-9), pro-BNP, N-terminal pro-atrial natriuretic peptide (NTpro-ANP), renin, myeloperoxidase, highly-sensitive C-reactive protein (hs-CRP), tumour necrosis factor receptor-1 (TNFR-1), interleukin-6, cystatin-C and neutrophil gelatinase-associated lipocalin (NGAL). Plasma biomarkers (total 22) were further categorized as either surrogates of myocardial interstitial fibrosis, LV cardiomyocyte stress/damage, myocardial hypertrophy, inflammation/oxidative stress, atrial wall stress/stretch or markers of renal dysfunction[20–22]. The assay ranges inclusive of upper and lower limits of quantitation, respective dilution factors and detailed analytical methods for each Luminex multiplexed plasma biomarker assay (Bristol Myers Squibb, Ewing Township, New Jersey, USA) are shown in S1 Table.

### Transthoracic echocardiography

Echocardiography was performed by accredited sonographers in accordance with American Society of Echocardiography guidelines using an iE33 system (Philips Medical Systems, Best, The Netherlands)[7]. LVEF was calculated using the biplane method or estimated visually where endocardial border definition was sub-optimal.

### CMR protocol

All scans were performed on a 3-Tesla platform (Siemens Skyra, Erlangen, Germany) with an 18-channel cardiac coil, as reported previously[4, 7]. This included standard long and short-axis cine imaging; basal, mid and apical short-axis pre- and post-contrast T1 mapping and LGE imaging. A total of 0.15mmol/kg of contrast (Gadovist, Bayer Healthcare, Berlin, Germany was administered.

### CMR analysis

*CVI42* software (Circle Cardiovascular Imaging, Calgary, Canada) was used for analysis, performed by a single operator (PK) blinded to clinical data. Papillary muscles and trabeculations were excluded from cavity volume when deriving ventricular volumes, EF and LV mass[4]. RV systolic dysfunction (RVD) was defined as RVEF <47% based upon normative data from the published literature utilizing the same technique[23] and our own healthy controls whereby the lower limit of RVEF was also 47%. The biplane method[24, 25], excluding the appendage and pulmonary veins was used to calculate left atrial (LA) volumes, LA ejection fraction, reservoir and conduit volumes. LA dilation[26] was defined as maximal LA volume indexed (LAVImax) >40ml/m^2^. Volumetric and mass data were indexed to body surface area.

LGE images were analyzed qualitatiavely for focal fibrosis, categorized as either absent or present and further quantified accordingly[4]. The mid-ventricular T1 maps were analyzed for ECV and iECV (ECV indexed to body surface area) as described previously[4]. Segments with MI or artefact were excluded but regions of focal non-MI fibrosis were included in the final ECV (and iECV) calculations. As reported previously[4], T1 mapping was not performed due to the sequence not being available in 55 (24%) consecutive CMR scans (HFpEF n = 44, HFrEF n = 7, controls n = 4). A further 4 patients with HFpEF had non-analyzable T1 maps.

#### Follow-up and endpoints

All subjects were followed up for the primary endpoint which was the composite of all-cause mortality or hospitalization for HF (defined as a hospital admission for which HF was the primary reason and requiring either diuretic, inotropic or intravenous nitrate therapy). Secondary outcomes were all-cause mortality and HF hospitalization. Outcome data were obtained from hospital records. In patients with multiple events, the time to first event was used as the censored outcome.

### Statistical analysis

SPSS v22 was used for statistical analyses. Normality for continuous data was assessed using the Shapiro-Wilk test, histograms and Q-Q plots. Normally distributed data are expressed as mean ±SD. Non-parametric data are expressed as median (25–75% IQR). Categorical data are expressed as absolute numbers or percentages. For comparison of normally distributed data between the 3 groups, the one-way ANOVA with Bonferroni correction was used. For comparison of non-normally distributed data, the Kruskal-Wallis test was employed. The Chi-square or Mann-Whitney U tests were to compare categorical data, as appropriate. For additional between group comparisons, multiple linear regression was used to adjust for clinical variables, where the dependent variable was the log transformed plasma biomarker of interest and independent variables included age, gender, body mass index (BMI) and co-morbidities [diabetes, hypertension, lung disease, atrial fibrillation (AF), chronic kidney disease (CKD), CAD). Binary logistic regression was undertaken with the same adjustments to test for differences in the presence of elevated troponin-I levels between HF groups. Spearman’s rank correlations were performed to detect any important associations with NTpro-ANP. Kaplan-Meier analysis was undertaken to calculate event rates. The Log-Rank test was used to test differences in survival curves. CMR assessments of intra-observer and inter-observer variability were undertaken at least 4 weeks apart (by PK and JRA), on a subset of 10 randomly selected patients (S1 Table).

## Results

**Fig 1:**
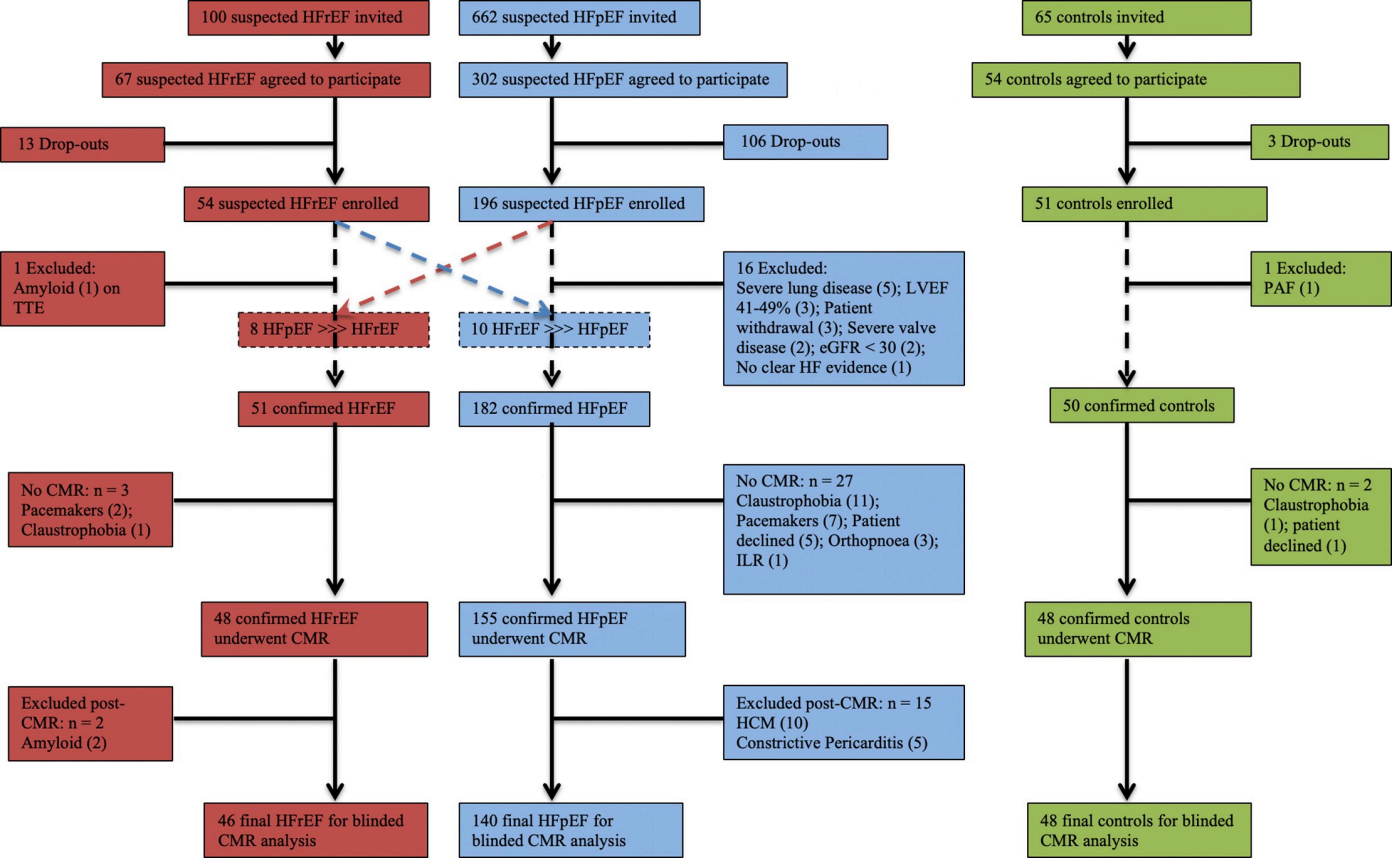
Study overview. Consort diagram illustrating recruitment and reasons for exclusion. CMR = cardiovascular magnetic resonance imaging; HCM = hypertrophic cardiomyopathy; HFpEF = heart failure with preserved ejection fraction; HFrEF = heart failure with reduced ejection fraction; ILR = implantable loop recorder; PAF = paroxysmal atrial fibrillation; TTE = transthoracic echocardiography.

The overall study consort diagram including reasons for exclusion are detailed in **Fig 1**. CMR was not performed due to the presence of pacemakers and such subjects were excluded from the final analysis in 2 out of 51 confirmed HFrEF and 7 out of 182 confirmed HFpEF. All subjects were recruited over a period of 26 months. The final participant was enrolled in April 2015. The final cohort comprised 234 subjects: HFpEF n = 140; HFrEF n = 46 and controls n = 48. The majority of HF patients were diagnosed and participated in the study as out-patients (HFpEF 57%; HFrEF 78%). All HF patients participating as in-patients had clinical evidence of heart failure (albeit deemed stable) at the time of their study visit and were on or had been receiving concomitant intravenous diuretic therapy.

### Baseline clinical characteristics

The baseline data are summarized in **Tables 1–3**. All 3 groups were evenly matched for age and sex distribution.

**Table 1:** Baseline clinical characteristics.

	HFpEF n = 140	HFrEF n = 46	Controls n = 48	p value
Age (years)	73±9	72±8	73±5	0.820
Male (%)	68 (49)	23 (50)	24 (50)	0.977
Heart rate (b.p.m)	70±14	67±16	68±10	0.308
Systolic BP (mmHg)	145±25	132±24 Δ*	151±24	0.001
Diastolic BP (mmHg)	74±12Δ	71±17 Δ	79±10	0.006
Body mass index (kg/m2)	34±7Δ	28±6*	25±3	<0.0001
Sinus Rhythm (%)	97 (69)Δ	37 (80)Δ	48 (100)	<0.0001
Atrial Fibrillation	43 (31)Δ	9 (20)	0 (0)	<0.0001
Diabetes (%)	75 (54)Δ	18 (39)Δ	0 (0)	<0.0001
Hypertension (%)	127 (91)Δ	25 (54)*	22 (46)	<0.0001
Angina (%)	23 (16)Δ	11 (24)Δ	0 (0)	0.003
Known Myocardial infarction (%)	16 (11)Δ	19 (41)Δ*	0 (0)	<0.0001
Coronary artery disease (%)	31 (22)Δ	23 (50)Δ*	0 (0)	<0.0001
Asthma or COPD (%)	24 (17)	9 (20)	3 (6)	0.134
Smoking (%)	75 (54)Δ	28 (61)Δ	17 (35)	0.033
Hypercholesterolaemia (%)	69 (49)	21 (46)	18 (38)	0.367
Peripheral Vascular Disease (%)	3 (2)	3 (7)	0 (0)	0.120
TIA or CVA (%)	19 (14)Δ	5 (24)Δ	0 (0)	0.025
Betablocker (%)	95 (68)Δ	41 (89)Δ*	2 (4)	<0.0001
ACEi or ARB (%)	120 (86)Δ	36 (78)Δ	10 (21)	<0.0001
Aldosterone antagonist (%)	43 (31)Δ	19 (41)Δ	0 (0)	<0.0001
Loop Diuretic (%)	113 (81)Δ	37 (80)Δ	0 (0)	<0.0001
NYHA III/IV (%)	43 (31)	12 (26)	NA	0.551
6 minute walk distance	180 (120–250)Δ	210 (165–290)Δ*	380 (350–440)	<0.0001
MLWHF score	49 (25–65)	36 (22–59)	NA	0.089
Sodium (mmol/L)	139±4	140±3	140±2	0.098
Urea (mmol/L)	9±4Δ	9 ± 4Δ	6±1	<0.0001
Creatinine (mmol/L)	89 (73–114.8)Δ	97 (77–128)Δ	71 (56.3–84.5)	<0.0001
eGFR (ml/min/m2)	68 (52–83)Δ	61 (48–77) Δ	92 (74–100)	<0.0001
CKD grade	Δ	Δ		<0.0001
1	27 (19)	6 (13)	26 (54)	-
2	54 (39)	17 (37)	20 (42)	-
3	59 (42)	23 (50)	2 (4)	-
Haemoglobin (g/L)	129±22Δ	134±24	140±15	0.003
Haematocrit (%)	38±6	40±7	41±4	0.071
BNP (ng/L)	135.6 (65.5–254.4)Δ	387 (178–634)Δ*	33 (24–44)	<0.0001

Δ P<0.05 for HFpEF or HFrEF versus controls

*P<0.05 for HFpEF vs HFrEF

Values are mean ± SD or n (%) or median (interquartile range). ACEi = angiotensin converting enzyme inhibitor; ARB = angiotensin II receptor blocker; BNP = B-type natriuretic peptide; CKD = chronic kidney disease; COPD = chronic obstructive pulmonary disease; CVA = cerebrovascular accident; eGFR = estimated glomerular filtration rate; NA = not applicable; NYHA = NewYork Heart Association; TIA = transient ischaemic attack

**Table 2:** Imaging characteristics by heart failure classification.

	HFpEF n = 140	HFrEF n = 46	Controls n = 48	p value
**Prior Chest Radiography**				
Pulmonary oedema (%)	97 (69)	31 (67)	NA	0.933
Raised CTR (%)	101 (72)	35 (76)	NA	0.362
Pleural effusion (%)	49 (35)	21 (46)	NA	0.138
Echo*				
E/E’	13±6Δ	15±5Δ*	9±3	<0.0001
**LA**				
*Overall including AF subjects*				
LAVImax (ml/m2)	43±19Δ	45±15Δ	30±7	<0.0001
LAVImin (ml/m2)	30±19Δ	33±16Δ	17±5	<0.0001
LAEF (%)	35±18Δ	28±15Δ	44±11	<0.0001
*Sinus rhythm subjects only*				
LAVImax (ml/m2)	36±14Δ	43±14Δ*	30±7	<0.0001
LAVImin (ml/m2)	21±12	29±12Δ*	17±5	<0.0001
LAEF (%)	44±14	32±12Δ*	44±11	<0.0001
**CMR**				
**LV**				
LVEDVI (ml/m2)	79±18	142±44Δ*	81±14	<0.0001
LVESVI (ml/m2)	35±10	106±44Δ*	34±8	<0.0001
LVEF (%)	56±5Δ	28±9Δ*	58±5	<0.0001
LVEDMI (g/m2)	52±15Δ	64±22Δ*	46±9	<0.0001
LV mass/LV volume	0.68±0.16Δ	0.47±0.15Δ*	0.57±0.09	<0.0001
**RV**				
RVEDVI (ml/m2)	80±19	86±27	83±15	0.212
RVESVI (ml/m2)	37±14	53±33Δ*	37±9	<0.0001
RVEF (%), median (range)	54 (27–74)	49 (20–72)Δ*	55 (47–70)	<0.0001
RV Dysfunction (%)	25 (19)Δ	21 (46)Δ*	0 (0)	<0.0001
**LA**				
*Overall including AF subjects*				
LAVImax (ml/m2)	53±25Δ	59±24Δ	35±12	<0.0001
LAVImin (ml/m2)	38±26Δ	44±24Δ	17± 8	<0.0001
LA reservoir volume indexed (ml/m2)	15±7	15±7	17±6	0.087
LA conduit volume indexed (ml/m2)	29±9	23±9Δ*	30±9	<0.0001
Dilated LA	90 (64)Δ	38 (83)Δ*	15 (31)	<0.0001
*Sinus rhythm subjects only*				
LAVImax (ml/m2)	43±17Δ	55±19Δ*	35±12	<0.0001
LAVImin (ml/m2)	26±13Δ	38±18Δ*	17±8	<0.0001
LA reservoir volume indexed (ml/m2)	17±6	17±6	17±6	0.957
LA conduit volume indexed (ml/m2)	28±8	22±9Δ*	30±9	<0.0001
LAEF (%)	41 ± 12Δ	33 ±12Δ*	51 ± 11	<0.0001
Dilated LA	48 (49)Δ	29 (78)Δ*	15 (31)	<0.0001
**LV Tissue characterization**				
ECV (%)	28±5Δ	31±8Δ*	25±3	<0.0001
iECV (ml/m^2^)	13.7±4.4Δ	18.1±7.1Δ*	10.9±2.8	<0.0001
LGE positive (%)	66 (47)Δ	41 (89)Δ*	5 (10)	<0.0001
LGE positive–MI (%)	23 (16)Δ	26 (57)Δ*	0 (0)	<0.0001
If MI, size of infarct as % of LV mass	3.0 (1.3–4.6)	9.8 (4.2–20.6)*	NA	<0.0001
LGE positive–non-MI	49 (35)Δ	19 (41)Δ	5 (10)	<0.0001
If non-MI, size of scar as % of LV mass	2.9 (1.4–6.5)	3.9 (2.2–7.7)	2.4 (0.6–3.6)	0.437

ΔP<0.05 HFpEF or HFrEF versus controls

*P<0.05 for HFpEF versus HFrEF

Values are mean ± SD or n (%). CTR = cardiothoracic ratio; ECV = extracellular volume; iECV = indexed ECV; LAEF = left atrial ejection fraction; LAVI = left atrial volume indexed to body surface area (maximal/minimal); LVEDMI = left ventricular end-diastolic mass indexed to body surface area; LVEDVI = left ventricular end-diastolic volume indexed to body surface area; MI = myocardial infarction; NA = not applicable; RVEF = right ventricular ejection fraction; RVEDVI = right ventricular end-diastolic volume indexed to body surface area

**Table 3:** Plasma biomarkers by heart failure classification.

	HFpEF n = 140	HFrEF n = 46	Controls n = 48	p value
**Interstitial fibrosis**				
ST-2 (ng/ml)	6386 (4956–8905)Δ	5117 (3912–8598)	5729 (4303–6940)	0.028
Galectin-3 (ng/ml)	7495 (5718–8998)Δ	7148 (6393–8859)Δ	5038 (4172–6104)	<0.0001
GDF-15 (ng/ml)	2248 (1546–3585)Δ	2117 (1509–3596)Δ	955 (665–1300)	<0.0001
Tenascin-C (ng/ml)	13.7 (10.8–17.3) Δ	13.1 (11.4–16.3) Δ	11.1 (8.9–12.9)	<0.0001
TIMP-1 (ng/ml)	1009 (755–1379)Δ	1013 (754–1271)Δ	662 (578–890)	<0.0001
TIMP-4 (ng/ml)	1.8 (1.4–2.4)Δ	1.7 (1.4–2.1)Δ	1.3 (1.2–1.6)	<0.0001
MMP-2 (ng/ml)	72.8 (58.6–86.8)Δ	69.7 (64.4–87.7)Δ	62.7 (56.2–68.7)	0.003
MMP-3 (ng/ml)	6.8 (4.6–10.3)Δ	7.8 (5.1–10.7)Δ	5.7 (3.2–7.5)	0.017
MMP-7 (ng/ml)	0.6 (0.4–1.0)Δ	0.8 (0.5–1.2)Δ	0.3 (0.2–0.5)	<0.0001
MMP-8 (ng/ml)	0.3 (0.2–0.4)Δ	0.3 (0.2–0.6)Δ	0.2 (0.1–0.3)	<0.0001
MMP-9 (ng/ml)	27.8 (19.5–56.8)Δ	36.6 (22.8–60.2)Δ	24.5 (15.5–37.8)	0.042
**LV Cardiomyocyte stress/damage**				
Elevated Troponin-I, ng/L (%)	33 (24)Δ	17 (37)Δ	0 (0)	<0.0001
BNP (ng/L)	136 (66–254)Δ	387 (178–634)Δ*	33 (24–44)	<0.0001
Pro-BNP (pg/ml)	1.6 (1.2–2.2)Δ	2.3 (1.6–4.3)Δ*	1.2 (1.1–1.4)	<0.0001
**Myocardial Hypertrophy**				
Renin (pg/ml)	389 (223–864)Δ	580 (259–1187)Δ	111 (61–202)	<0.0001
**Inflammation/oxidative stress**				
Myeloperoxidase (ng/ml)	212 (160–262)Δ	203 (147–283)Δ	153 (130–178)	<0.0001
hs-CRP (ng/ml)	43169 (14992–78805)Δ	24604 (6246–67468)Δ	6914 (3531–17393)	<0.0001
TNFR-1 (ng/ml)	5.4 (4.1–7.8)Δ	5.6 (3.9–7.8)Δ	3.2 (2.7–3.7)	<0.0001
Interleukin-6 (pg/ml)	4.0 (3.3–5.1)Δ	3.7 (3.2–5.3)Δ	2.9 (2.5–3.2)	<0.0001
**Atrial stress/stretch**				
NTpro-ANP (pg/ml)	6443 (4362–8511)Δ	7814 (6226–10097)Δ*	4019 (3362–4475)	<0.0001
**Renal markers**				
Cystatin C (ng/ml)	776 (686–989)Δ	811 (676–996)Δ	586 (526–648)	<0.0001
NGAL (ng/ml)	44.6 (32.9–58.5)Δ	48.6 (33.1–63.4)Δ	26.4 (21.2–34.3)	<0.0001

Δ P<0.05 for HFpEF or HFrEF versus controls

*P<0.05 for HFpEF vs HFrEF

Values are median (IQR) or n (%). GDF-15 = growth differentiation factor-15; hs-CRP = highly-sensitive C-reactive protein; MMP = matrix metalloproteinases; NGAL = neutrophil gelatinase-associated lipocalin; NTpro-ANP = N-terminal pro-atrial natriuretic peptide; ST2 = suppression of tumorigencity-2; TIMP = tissue inhibitor of metalloproteinase; TNFR-1 = tumour necrosis factor receptor-1

#### Controls

Hypertensive controls were older (75±6 vs 71±3 years, p = 0.006), had a higher proportion of smokers, hypercholesterolaemia and exhibited lower exercise capacity (360 (325–423) vs 410 (378–456)m, p = 0.007) compared to non-hypertensive controls. The presence of hypertension correlated with the finding of focal fibrosis in controls (r = 0.328, p = 0.023). Focal fibrosis (non-ischaemic in all cases) was observed in a small minority of hypertensive controls (18%) and absent in non-hypertensive controls. There were no other significant clinical, imaging or plasma biomarker differences between the control groups (also see **S3–S5 Tables**).

#### HF versus controls

Compared to controls, HF patients had a significantly greater prevalence of CAD and diabetes, poorer renal function and higher LV filling pressures (i.e. higher E/E’ and BNP). Exercise capacity was markedly worse in HF patients.

#### HFpEF versus HFrEF

Compared to HFrEF, patients with HFpEF had less CAD, higher body mass index, less severe diastolic dysfunction (i.e. lower BNP and E/E’), more atrial fibrillation (AF) (31% versus 20%) and lower 6MWT distance (180m versus 210m; p = 0.038). The MLHF score tended to be higher in HFpEF (p = 0.089) and NYHA class was similar between groups. Abnormal chest X-ray findings were similarly prevalent in both HF groups (**Table 2**).

### CMR

#### LV parameters

**Fig 2:**
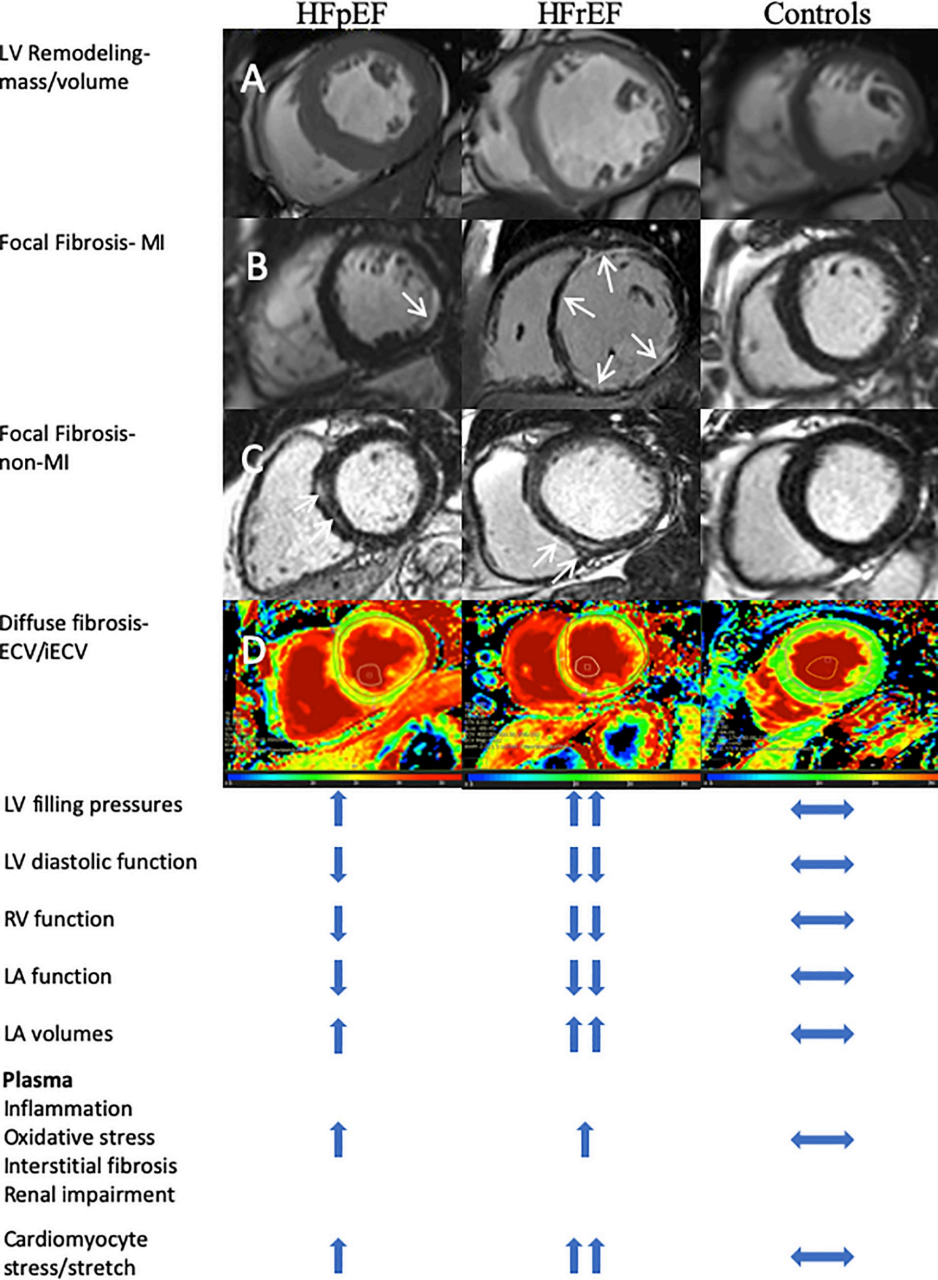
Summary of directional change of CMR and plasma biomarkers profiles compared to controls. CMR images at the top of the illustration. Panel A: mid-ventricular end-diastolic cines showing concentric remodeling in HFpEF and eccentric remodeling in HFrEF; Panel B: Late gadolinium enhancement images showing (white arrows) small, sub-endocardial ischemic fibrosis in HFpEF and more extensive ischemic fibrosis involving multiple segments in HFrEF; Panel C: Late gadolinium enhancement images showing (white arrows) non-ischemic fibrosis in HFpEF and HFrEF; Panel D: Post-contrast mid-ventricular extra-cellular volume colour maps showing increased diffuse fibrosis in both HFpEF and HFrEF.

Compared to controls, LVEF was marginally lower in HFpEF, albeit preserved overall (p = 0.019). LV volumes were similar but HFpEF patients exhibited higher LV mass and more concentric remodeling (higher mass/volume ratio). In comparison to HFpEF, patients with HFrEF had marked reductions in LVEF and substantially higher LV volumes and mass but with a reduction in mass/volume ratio indicative of adverse eccentric remodeling. Examples of typical CMR LV characteristics (plus additional imaging and plasma profiles) of the HF groups are illustrated in **Fig 2**.

#### RV parameters

RVEF was lower in HFrEF compared to the other groups. RVD was more prevalent in HFrEF (46%) compared to HFpEF (19%) and was also associated with greater remodeling (increased RV end-systolic volumes) compared to HFpEF and controls.

#### LA parameters

Across the cohort and irrespective of cardiac rhythm, HF patients had higher LA volumes and a greater proportion of dilated atria (p<0.0001). In sinus rhythm, LAEF was lower in HF patients compared to controls and lowest in HFrEF (p<0.0001). LA conduit function was depressed in HFrEF compared to HFpEF or controls.

#### Tissue characterization

Qualitatively, focal fibrosis was more prevalent in both HF groups compared to controls and was commonest in HFrEF (controls 10%, HFpEF 47%, HFrEF 89%, p<0.0001). Likewise, similar trends were observed for ischemic (0%; 16%; 57%) and non-ischemic fibrosis (10%; 35%; 41%), p<0.0001 for both. A small subset of HF patients exhibited both patterns of focal fibrosis: HFpEF n = 4 and HFrEF n = 5. The size of MI expressed as a percentage of LV mass, was larger in HFrEF (9.8% vs 3%, p<0.0001) compared to HFpEF. However, there was no significant difference in the extent of non-ischemic fibrosis (p = 0.179) between HF groups. Diffuse fibrosis (ECV, iECV) was also higher in both HF groups compared to controls and greatest in HFrEF: controls (25%, 10.9±2.8), HFpEF (28%, 13.7±4.4), HFrEF (31%, 18.1±7.1); p<0.0001.

#### Inter-observer and intra-observer assessments

Data quantification for all CMR parameters are shown in **S6 Table**. All intra-observer agreements were excellent (co-eficient of variations <10%) and universally better than for inter-observer agreements. The majority of inter-observer agreements remained excellent albeit LV end-systolic volumes, RV end-systolic volumes and RV ejection fraction fared worse (but still good).

### Plasma biomarkers

**Fig 3:**
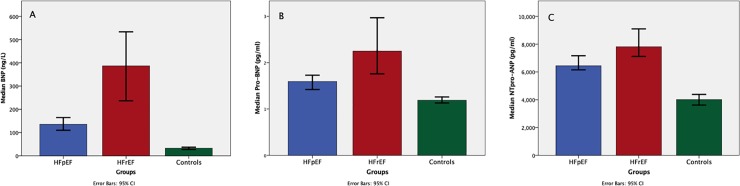
Plasma biomarkers that were different between HFpEF and HFrEF: Natriuretic peptides. BNP = B-type natriuretic peptide; Pro-BNP = pro-B-type natriuretic peptide; NTpro-ANP = N-terminal pro-atrial natriuretic peptide; p<0.05 for all.

All plasma biomarkers tested in the panel except ST2 displayed higher levels in both HF groups compared to controls. The majority of biomarkers were not different between HFpEF and HFrEF. The exceptions were the natriuretic peptides (**Fig 3**) and Troponin-I. BNP, pro-BNP and NTpro-ANP levels were highest in HFrEF. After adjusting for potential confounders including age, sex, BMI, diabetes, hypertension, lung disease, AF and CKD, these differences (or similarities) in plasma biomarkers between HFpEF and HFrEF persisted (see S2 Table) with the exception of MMP-8. Likewise, following binary logistic regression and adjustments for the same variables listed above, the presence of troponin-I elevation was different between the HF groups (odds ratio 2.536; 95% CI 1.033–6.230; p = 0.042).

The presence of focal fibrosis on LGE correlated with the following plasma biomarkers of interstitial fibrosis: galectin3, GDF-15, MMP-3, MMP-7, MMP-8 and cardiomyocyte stress/stretch (BNP, pro-BNP, NTproANP); p<0.05 for all. ECV (diffuse fibrosis) also correlated with plasma interstitial fibrotic markers (GDF-15, Tenascin-C, MMP-2, MMP-3, MMP-7) and cardiomyocyte stress/stretch (BNP, proBNP, NTproANP); p<0.05 for all. The results of correlations of imaging parameters reflecting fibrosis with plasma biomarkers are shown in **S7 and S8 Tables**.

NTpro-ANP strongly correlated with: BNP r = 0.675; minimal LA volume indexed (LAVImin) r = 0.608; LAVImax r = 0.580; LAEF r = -0.506; p<0.0001 for all (**S1 Fig**). Troponin elevations were only observed in the HF cohorts: HFpEF 24%; HFrEF 37%. Hs-CRP levels were highest in HFpEF but not significantly different compared to HFrEF (p = 0.082).

#### Sub-group analysis of HF patients excluding known CAD and/or MI on LGE

Following exclusion of HF subjects with known CAD or with evidence of MI on LGE (n = 43 HFpEF, n = 28 HFrEF), the higher prevalence of non-ischemic fibrosis in HFrEF compared to HFpEF remained (72% vs 38%, p = 0.006). Furthermore, the trends for key imaging differences in these sub-groups between HFrEF and HFpEF remained the same with respect to LVEF, LV remodeling patterns, RVD, LA dysfunction and iECV. Likewise, higher levels of cardiomyocyte stress/damage plasma biomarkers were again observed in HFrEF relative to HFpEF (see **S9–S11 Tables**).

#### Endpoints

During median follow-up (overall 1446 [1243–1613]; HFpEF 1429 [1157–1567]; HFrEF 1492 [1392–1545] days), the proportion of events were similar for HFpEF (67 composite events [48%], 45 HF hospitalizations, 22 deaths) compared to HFrEF (20 composite events [43%], 14 HF hospitalizations, 6 deaths); p = 0.606. The event rates and time to first events did not differ significantly between HFrEF and HFpEF for both primary (Log-Rank p = 0.784) and secondary outcomes (all-cause mortality Log-Rank p = 0.372; HF hospitalization Log-Rank p = 0.705). Kaplan-Meier survival curves stratified according to both HF groups are shown in **S2 Fig**. There were no events in the control group.

## Discussion

Our phenotyping study provides important insights into the clinical and pathophysiological profiles of HFpEF relative to HFrEF and controls. Firstly, our study re-affirms the clinical heterogeniety of HFpEF and its association with adverse outcomes and exercise incapacity. Secondly, striking differences in CMR parameters, underscored by plasma biomarkers were seen in both HF groups compared to controls. Thirdly, imaging differences of cardiac structure and function were clearly evident between HFpEF and HFrEF at the chamber levels (LV, RV and LA) as well as at the LV tissue level (focal and diffuse fibrosis). Finally, plasma markers of inflammation, fibrosis, myocardial hypertrophy and renal dysfunction were consistently elevated in both HF phenotypes but only markers of cardiomyocyte damage/loss were higher in HFrEF compared to HFpEF.

### Clinical phenotypes and characterization

Our HFpEF cohort was characterized by a high prevalence of both cardiovascular (hypertension, CAD, diabetes, AF) and non-cardiovascular disease (obesity, renal dysfunction, lung disease, anaemia), consistent with prevailing literature[3]. In addition, compared to HFrEF, HFpEF patients had higher BMI, greater proportion of hypertension and AF but less CAD as also noted previously[2, 3]. Both HF groups displayed markedly reduced exercise capacity and quality of life, consistent with the diagnoses[27]. Unlike prior literature however[27], exercise capacity was lower and HF symptoms tended to be higher in HFpEF compared to HFrEF in our cohort. Possible explanations for this include the contribution of the greater co-morbidity burden seen in HFpEF[28], as well as vascular stiffening and reduced aortic distensibilty[29], measures not yet assessed in our cohort but which may further impact upon 6MWT distance.

### Imaging and plasma biomarkers

As expected we observed changes in LV structure and function (systolic and diastolic) across both HF cohorts compared to controls. However, the pattern of these changes was markedly different between HFpEF and HFrEF. The lower LVEF in HFpEF (albeit preserved overall) compared to controls has previously been observed with TTE and was thought to reflect mildly reduced contractile function, subtle systolic abnormalities and impaired longitudinal function[30]. In our HFpEF cohort, both CMR and plasma changes further explain the likely aetiology of such disturbances, with MI noted in a significant minority (16%), non-ischemic focal fibrosis in nearly half and an increased proportion of patients having evidence of cardiomyocte damage/loss (raised Troponin-I). In contrast, LVEF was markedly reduced in HFrEF and was associated with higher focal fibrotic burden (both MI and non-ischemic) and an even greater proportion with elevated Troponin-I.

In agreement with previous reports[2, 3], we also observed different patterns of adverse remodeling in HFpEF (concentric) and HFrEF (eccentric). Parameters previously implicated in the pathophysiology of adverse remodeling in HF were also found to be elevated in our study: interstitial fibrosis (CMR and plasma), inflammation, oxidative stress, myocardial hypetrophy, RAAS activation, cardiomyocyte damage and renal dysfunction[20–22, 31]. Of these, only markers of cardiomyocyte stretch/damage differed between HF groups.

Recently, alternative paradigms for HFpEF and HFrEF have been proposed[32], postulating that prevalent co-morbidities in HFpEF induce a pro-inflammatory state which is the predominant pathophysiological mechanism influencing LV remodeling. Systemic inflammation induces microvascular endothelial dysfunction, which in turn limits nitric oxide bioavailablity, decreasing protein kinase G activity, promoting both cardiomyocyte hypertrophy (concentric remodeling) and stiffening (titin hypophosphorylation), with resultant myofibroblast-induced interstitial fibrosis. In contrast, predominant cardiomyocyte damage/loss resulting in focal (replacement) fibrosis is thought to be the main driver behind chamber dilation and eccentric remodeling in HFrEF.

However, the data supporting the differential role of inflammation and cardiomyocyte damage in HFpEF and HFrEF has been conflicting to. While some studies[22, 33] have shown contrasting levels of inflammatory markers between HFpEF and HFrEF, others have shown similar levels in both HF sub-types, as observed in our study[34]. We also noted a clear signal for cardiomyocyte damage as the predominant mechanism in HFrEF with higher levels of natriuretic peptides and with Troponin-I. Furthermore, CMR detected focal fibrosis was also higher in HFrEF. Diffuse fibrosis is thought to represent vulnerable myocardium prior to the transitory phase towards focal, irreversible (replacement) fibrosis development. Our finding of more prevalent focal fibrosis in HFrEF is also a likely reflection of this phenomenon. Only one prior CMR study[35] has compared diffuse fibrosis between HFpEF and HFrEF with similar findings to the present study.

Diastolic dysfunction noted in HF is primarily governed by myocardial hypertrophy (and stiffness), which in turn is regulated at the tissue level by alterations in cardiomyocytes and the extracellular matrix[32]. Given that the degree of fibrosis, and consequent remodeling, is vastly different between both HF groups, surrogates of diastolic dysfunction i.e. natriuretic peptides, E/E’ and LA volumes were unsurprisingly higher in HFrEF. Furthermore, end-diastolic wall stress in HFpEF is less pronounced[36], reducing the stimulus for natriuretic peptide secretion. In addition, the greater prevalence of obesity in HFpEF may further blunt natriuretic peptide levels[36].

### Right ventricular dysfunction

A wide range of prevalence for RVD, primarily derived from TTE data has previously been reported in HFpEF (4–44%), utilizing variable definitions of HFpEF e.g. LVEF ≥45% and different diagnostic thresholds for RVD (tricuspid annular plane systolic excursion, fractional area change and RVEF)[37, 38]. There has also been conflicting data as to whether RVD prevalence differs between HFpEF and HFrEF[38]. In HFpEF, only 2 CMR studies have analyzed RV performance and both lacked control groups. In the first study[39] (n = 142) significant RVD was defined semi-quantitatively as the presence of least moderate RVD (prevalence 12%). The second study[40] (n = 171) using a RVEF cut-off of <45% reported RVD prevalence of 19%. To our knowledge, no prospective CMR studies have compared RVD in HFpEF and HFrEF.

Our study confirms that RVD is present in a significant minority of HFpEF and is also more prevalent in HFrEF compared to HFpEF, based upon our own internal reference controls. RVD may be part of the natural aetiological profile in HFpEF whereby biventricular remodeling often co-exists, even in early stages[41] or as a marker of prognosis[37]. The high concurrent burden of lung disease, CAD and diastolic dysfunction have all previously been implicated in the aetiology of RVD in HFpEF[37]. Our findings of a greater degree of RVD in HFrEF compared to HFpEF is likely explained in part by the higher proportion of CAD (ischaemia and MI) in our HFrEF group which is intrinsically linked to impaired RV contractility[38]. Furthermore, impaired LV contractility is also known to indirectly impair RV performance[42].

### LA dysfunction and remodeling

In our study, compared to controls, both HF groups displayed more adverse LA remodeling and dysfunction, irrespective of AF. Our findings are further supported by the differential levels of NTpro-ANP (a marker of atrial as well as cardiomyocyte stress/stretch) observed across all groups and its strong correlations with LAEF and LA volumes.

Our work is additive to the growing evidence base implicating these parameters in HF[43]. Impaired LA function has also been noted in antecedent conditions of HF (e.g. diabetes, hypertension) even in the presence of normal LA size[44]. Similar to our findings, lower LAEF has been previously shown in HFpEF compared to hypertensive subjects with LV hypertrophy[45]. Furthermore, a trend towards worse LAEF in HFrEF when compared to HFpEF has also been reported using TTE[46]. In that study, analogous to structural changes in the LV, HFrEF displayed more eccentric LA remodeling whilst HFpEF was characterized by higher LA wall stress. In our study, worsening LA dilation was observed in HFrEF compared to HFpEF, even in the absence of AF. Furthermore, the differing degrees of LAEF reduction and LA remodeling seen in our HF groups mirrored the degree of adverse remodeling in the LV, suggesting indirect upstream LA consequences. This hypothesis is further supported by the strong correlation of NTpro-ANP, a more specific marker of LA stress/stretch than BNP.

### Implications

HFpEF has been the subject of ongoing debate as to whether it truly exists or it is just a collection of co-morbidities in elderly subjects that ultimately drive symptoms and outcomes[2, 3]. Our study provides supportive evidence that HFpEF is a clinical entity distinct from controls and HFrEF and is characterized by pathophysiological disturbances across a range of both CMR and plasma biomarker measures, even when accounting for the influence of age, sex and additional comorbidity.

Our group has previously shown[7] that in those with ‘suspected’ HFpEF who underwent standard evaluation with echocardiography, utilizing CMR identifies new (27%), previously undiagnosed clinical pathologies (e.g. HCM, constrictive pericarditis) which may alternatively account for symptoms but also are independently predictive of prognosis. In this current study, following exclusion of such pathologies, we were able to study a ‘purer’ cohort of HFpEF.

The structural and functional changes observed in our HFpEF cohort may also carry prognostic relevance and have ramifications for future clinical trial design and therapies. Worse outcomes have been previously shown in the presence of adverse LV remodeling[47], focal fibrosis[48] and RVD[39, 40] in HFpEF. We have also shown that diffuse fibrosis (iECV and ECV)[49] and LA dysfunction[24] are independently related to prognosis in our HFpEF cohort. The distinct patterns of LV remodeling seen in the HF groups and the greater degree of cardiomyocyte damage/stress seen in our HFrEF cohort also provide potential insights into the failure of traditional HFrEF vasodilator therapies when tested in HFpEF clinical trials. The slope of the end-systolic pressure-volume relationship (or end-systolic LV elastance), a measure of contractility is influenced by chamber size. In HFpEF, elastance is increased and heightens sensitivity to volume changes resulting in substantial vasodilator-induced blood pressure (BP) reductions. In HFrEF however, elastance is diminished and similar therapy improves stroke volume without such BP drops[50]. Finally, CMR potentially enables HFpEF to be further sub-categorised into clinical phenotypes e.g. ischemic versus non-ischemic and pathophysiological sub-types enabling more targeted therapies as recently proposed[2]. Integrating plasma markers not only provides supportive measures for CMR derangements, but could also serve as potential biomarkers to assess treatment response[31]. LA dysfunction is a potential treatment target in HFpEF[51] and the use of CMR-derived LAEF and NT-proANP may serve as useful outcome measures in this setting. Furthermore, the association of plasma biomarkers of interstitial fibrosis and cardiomyocyte stress/damage with LGE and ECV in our study may be of potential benefit where fibrosis is the focus of treatment strategies in HFpEF[52]. Our study adds to the growing data for the associations of plasma biomarkers with CMR measures of fibrosis. MMP-2, galectin-3[53] and BNP[54] have previously been show to correlate with ECV in HFpEF. In severe aortic stenosis patients[55], phenotypically similar to HFpEF, both ECV and LGE have also been shown to correlate with NTpro-BNP. Even in subjects free of cardiovascular disease, NTpro-BNP also correlated with ECV in the MESA study[56].

### Strengths and limitations

We tested an extensive array of biomarkers reflecting individual domains which provides a more unbiased approach to discriminating key pathophysiological differences between our cohorts. Furthermore, such changes in biomarker profiles corroborate the CMR findings. While imaging parameters have been used to characterise HFpEF previously, these have largely been from echocardiographic data. Prior CMR studies in this setting have not only been sparse but largely confined to assessment of individual characteristics such as the LV, RV or diffuse fibrosis and lacking controls or HFrEF groups for comparison. Our study is the first to undertake a comprehensive and combined assessment of all of the above parameters as well as quantifying focal fibrosis, using a novel metric of diffuse fibrosis (iECV) and undertaking RV and LA volumetric and EF measurements across all 3 groups.

This is a single centre, observational study with possible selection bias. Therefore, the results should be confirmed in additional populations. We do not have additional information regarding the duration of HF. A small proportion of subjects did not undergo CMR due to the presence of pacemakers. At the time of study conduct, our centre was not implanting CMR conditional devices. We recognize that the unequal group sizes and higher number of patients in the HFpEF group are potentially confounding. However, we minimized the effects of age and sex by matching for these variables between the groups. Furthermore, we accounted for additional comorbidities in our statistical analysis. We deliberately chose a higher HFpEF sample size in our study since HFpEF is widely recognized to be a more heterogenous entity relative to HFrEF[1]. We did not account for additional co-morbidities such as prior chemotherapy/radiotherapy or other systemic conditions which may have influenced plasma biomarker levels as well as imaging markers of fibrosis.

Coronary angiography was only performed if clinically indicated. Furthermore, the HFrEF group had a higher proportion who underwent coronary angiography (24/46) compared to HFpEF (27/140). Therefore, we cannot exclude a higher prevalence of undiagnosed CAD in the HFpEF group which may account for some differences in plasma biomarker levels between the groups. Prior literature has consistently revealed a higher comorbidity burden in HFpEF compared to HFrEF[8, 11]. Indeed, obesity and hypertension were more prevalent in our HFpEF group compared to HFrEF. However, whilst the proportion of additional comorbidities such as diabetes and AF were also greater in HFpEF compared to HFrEF, this did not reach statistical significance, likely a reflection of our sample size. The lack of differences in prevalent lung disease and CKD between our HF groups however is most likely a consequence of our inclusion criteria and some selection bias. We excluded patients with severe lung disease which may alternatively explain HF symptoms. Furthermore, severe renal dysfunction (i.e. eGFR <30) was a contraindication for CMR evaluation with contrast administration. While the higher BMI levels in our HFpEF cohort compared to HFrEF are consistent with prior observations[8, 11, 57], the mean BMI [28] in our HFrEF group relative to healthy controls was also elevated. However, BMI tends to lessen with advancing and indeed more severe HFrEF. Our HFrEF cohort comprised relatively stable, ambulant patients who probably had less severe HF as reflected by only a small minority exhibiting NYHA class III/IV (26%). The mean BMI and proportion of NYHA III/IV seen in our HFrEF group was also similar to that observed in recent large, contemporary randomised control clinical trials of HFrEF (PARADIGM-HF[58], DAPA-HF[59]). Furthermore, our controls were relatively lean with only a handful being obese (8%) likely exaggerating the differences relative to HFrEF. As the HFrEF cohort were age and sex-matched to the HFpEF population and were not consecutively enrolled, they may not be representative of the general population with HFrEF, albeit similar in demographics to recent large phase III clinical HFrEF trials as reported above[58, 59].

We measured Troponin-I with a validated assay routinely used by our institution at the time of our study. We do recognise however that this assay has been superceded by more highly sensitive measures of troponin-I with lower limits of detection and which have shown increased troponin-I levels above the 99th percentile upper reference limit in the majority of HF patients[60–62]. Utilising these newer assays may have enabled better troponin-I characterisation and profiling between our groups. In our study, ST2 levels were higher in HFpEF compared to HFrEF but not of statistical significance. In contrast, prior HF studies[63–66] have demonstrated higher ST2 levels in HFrEF compared to HFpEF, each using similar assay methodologies (ELISA) but different to our technique of ST2 quantification. This discrepancy likely represents the possible effects of unequal sample sizes of the groups in our cohort, assay technique and the heterogeneous nature of HFpEF itself, albeit our HFpEF sample size is the largest to date and the technique used to measure ST2 levels has also been studied in additional HFpEF populations[18, 19].

Study participation as in- or out-patients with differing fluid status, or recruitment of in-patient HF patients who were receiving or had been on preceding intravenous diuretic therapies may alternatively have contributed to differing plasma biomarker levels. Patient enrolment into our study had already been completed by the time of publication of the latest ESC diagnostic HF guidelines in 2016[1]. Unlike latest ESC guidance[1] however, our inclusion criteria did not require the presence of diastolic dysfunction for HFpEF diagnosis nor elevated natriuretic peptide levels. However, diastolic dysfunction is often absent at rest in approximately a third of such patients[9]. All patients with a diagnosis of HFpEF in our cohort had a history of (or at the time of their study visit) signs and symptoms of HF as per ESC diagnostic guidelines. In addition, at screening, a diagnostic label of HFpEF was already made by a consultant Cardiologist either at a prior outpatient clinic visit or following a prior hospitalization episode. Only a very small minority of HFpEF patients in our cohort (12%) did not have elevated BNP levels as per ESC guidelines (≥35 pg/ml). However, natriuretic peptide levels well below ESC diagnostic thresholds have previously been observed in a significant minority (18%) of invasively proven HFpEF[10], albeit we recognize left and right heart catheterization data is lacking in our study. In our sub-group of patients with non-elevated BNP defined as HFpEF, a high proportion of obesity (BMI ≥30 kg/m2 in 81%) was observed which may additionally account for the supressed BNP levels[67]. Furthermore, our data clearly provides compelling evidence (additional natriuretic peptides) that our HFpEF cohort truly had HF and the high event rates are similar to that of previous outcome studies in HFpEF and indeed our HFrEF cohort. Our control group also included hypertensive subjects and was therefore not totally free of cardiovascular disease but the rationale for including such controls was to better understand the mechanisms leading to HFpEF. If anything, this is likely to have potentially underestimated the differences between HFpEF (and HFrEF) and control groups.

## Conclusions

Compared to HFpEF, HFrEF has worse LV, LA and RV contractile function and more prevalent fibrosis (focal and diffuse). Both HFpEF and HFrEF are associated with similar adverse outcomes. While inflammation is common in both HF phenotypes, cardiomyocyte stretch/stress is greater in HFrEF suggesting that HFpEF is a distinct clinical entity.

## Supporting information

S1 Table Plasma biomarker analytical characteristics and methodology. (DOCX)

S2 Table Between group comparison for plasma biomarkers following adjustment for covariates. (DOCX)

S3 Table Baseline clinical characteristics of hypertensive versus non-hypertensive controls. (DOCX)

S4 Table Imaging characteristics of hypertensive versus non-hypertensive controls. (DOCX)

S5 Table Plasma biomarker profiles of hypertensive versus non-hypertensive controls. (DOCX)

S6 Table Intra-observer and inter-observer assessments for CMR parameters. (DOCX)

S7 Table Significant associations of the presence of focal fibrosis on late gadolinium enhancement imaging with other plasma biomarkers. (DOCX)

S8 Table Significant associations of diffuse fibrosis (extracellular volume) on CMR with other plasma biomarkers. (DOCX)

S9 Table Baseline clinical characteristics of heart failure sub-groups following exclusion of known coronary artery disease and/or MI on LGE. (DOCX)

S10 Table Imaging characteristics of heart failure sub-groups following exclusion of known coronary artery disease and/or MI on LGE. (DOCX)

S11 Table Plasma biomarker profiles of heart failure sub-groups following exclusion of known coronary artery disease and/or MI on LGE. (DOCX)

S1 Fig Correlations of NTpro-ANP. Scatter plots illustrating the relationship between NTpro-ANP and: A) BNP B) minimum left atrium volume indexed–LAVImin C) maximum left atrium volume indexed—LAVImax D) left atrial ejection fraction.
(TIFF)

S2 Fig Kaplan-Meier survival analysis. Survival curves stratified according to heart failure groups for the primary endpoint: composite of all-cause mortality or hospitalization for HF (left panel); all-cause mortality (middle panel); HF hospitalization (right panel).
(TIFF)
