# Supplementary material for: Characterizing heart failure with preserved and reduced ejection fraction: An imaging and plasma biomarker approach
Source: PLoS One. 2020 Apr 29;15(4):e0232280. doi: 10.1371/journal.pone.0232280 (PMC7190371; doi:10.1371/journal.pone.0232280)
Supplement: S1 Table — (DOCX) [file pone.0232280.s001.docx]

**S1 Table 1: Plasma biomarker analytical characteristics**

| **Plasma Biomarkers analysed by BMS** | **Upper Limit of Quantitation (pg/ml)** | **Lower Limit of Quantitation (pg/ml)** | **Dilution Factor** |
| --- | --- | --- | --- |
| **Interstitial fibrosis** | | | |
| ST-2 | 283575 | 553.86 | 1 |
| Galectin-3 | 1345 | 2.63 | 40 |
| GDF-15 | 4710 | 9.2 | 1 |
| Tenascin-C | 1285 | 2.51 | 40 |
| TIMP-1 | 11453.3 | 85.7 | 400 |
| TIMP-4 | 1075 | 2.1 | 1 |
| MMP-2 | 69665 | 136.06 | 40 |
| MMP-3 | 1345 | 2.63 | 40 |
| MMP-7 | 46200 | 90.23 | 1 |
| MMP-8 | 25535 | 49.87 | 1 |
| MMP-9 | 4375 | 8.54 | 40 |
| **LV Cardiomyocyte stress/damage** | | | |
| Pro-BNP | 9505 | 18.56 | 1 |
| **Myocardial Hypertrophy** | | | |
| Renin | 12185 | 23.8 | 1 |
| **Inflammation/oxidative stress** | | | |
| Myeloperoxidase | 5961.7 | 39.5 | 400 |
| hs-CRP | 8942.5 | 67.7 | 400 |
| TNFR-1 | 7130 | 13.93 | 1 |
| Interleukin-6 | 75 | 0.15 | 1 |
| **Atrial stress/stretch** | | | |
| NTpro-ANP | 11715 | 22.88 | 1 |
| **Renal markers** | | | |
| Cystatin C | 24775.7 | 21.3 | 400 |
| NGAL | 19060 | 37.23 | 40 |
| BMS= Bristol-Myers Squibb; GDF-15 = growth differentiation factor-15; hs-CRP = highly-sensitive C-reactive protein; MMP = matrix metalloproteinases; NGAL = neutrophil gelatinase-associated lipocalin; NTpro-ANP = N-terminal pro-atrial natriuretic peptide; ST2 = suppression of tumorigencity-2 ; TIMP = tissue inhibitor of metalloproteinase; TNFR-1 = tumour necrosis factor receptor-1 | | | |

**BMS plasma biomarker analytical methods**

Luminex® xMAP technology^1^ multiplexed arrays enable the simultaneous detection and quantification of up to 100 biomarkers in a single plasma sample. The technology comprises polystyrene bead arrays encoded with differing intensities of red/infrared dyes and coated with a specific-capture antibody against a biomarker of interest. Spectral signatures of the beads and the associated biomarkers are identified following interrogation of the beads by two lasers. The R-phycoerythrin (RPE) labelled secondary antibody against the specific biomarker allows for the quantitation of the biomarker.

Luminex reagents for all the plasma biomarkers described in the above Table were purchased from R&D Systems (Minneapolis, MN, USA) which also provided corresponding lyophilized protein standards. 50μL of plasma samples were stored at -70^0^C in a 96-well format prior to use. Samples were diluted according to manufacturer’s protocol (see Table and below) immediately prior to assaying.

For validation purposes, the dynamic range of each biomarker was determined by the lowest and highest standard points with acceptable coefficient of variation (%CV) and accuracy. Accuracy was defined by spike and recovery study in human matrix with the percentage recovery having to be within 70 – 130% of pre-defined values. All plasma biomarkers studied in our cohort had %CV ≤30%. For determination of plasma biomarker concentrations, the following methods and protocols were followed:

a) incubation with capture antibodies and RPE labeled detection antibodies

b) washing of Luminex beads in the assay plates

c) measurement on a Luminex Bio-Plex 3D Reader [(Bio-Rad, Hercules, CA, USA); a minimum of 50 bead events per biomarker was required]

d) raw data was measured as mean fluorescence intensity (MFI)

e) a 4-parameter logistic curve fit generated from Bio-Plex software using triplicate standard curves for each biomarker in the same assay plate was used to derive the concentration of each biomarker (pg/mL)

f) each standard curve had 7 standard concentrations based on 1 to 4 serial dilutions

The lower and upper limits of quantitation (LLOQ, ULOQ) for all plasma biomarkers measured with the above methods are shown in the Table above. The normal range lies between those boundaries. Samples were diluted such that all plasma biomarker levels fall into that range. The lower limit of detection (LOD) equated to the LLOQ in our cohort since we discarded any values that extrapolated below the LLOQ and replaced it with the actual LLOQ value.

Reference:

1. Luminex® Assays and Luminex High Performance Assays: R&D Systems. 2017. https://www.rndsystems.com/products/luminex-assays-and-high-performance-assays
