# Supplementary material for: Characterizing heart failure with preserved and reduced ejection fraction: An imaging and plasma biomarker approach
Source: PLoS One. 2020 Apr 29;15(4):e0232280. doi: 10.1371/journal.pone.0232280 (PMC7190371; doi:10.1371/journal.pone.0232280)
Supplement: S2 Table — (DOCX) [file pone.0232280.s002.docx]

**S2 Table S2: Between group comparison for plasma biomarkers following adjustment for covariates**

| **Multiple Linear Regression** | |
| --- | --- |
| **Plasma Biomarkers** | **HFpEF vs HFrEF**  **p value** |
| **Interstitial fibrosis** | |
| ST-2 | 0.658 |
| Galectin-3 | 0.873 |
| GDF-15 | 0.453 |
| Tenascin-C | 0.960 |
| TIMP-1 | 0.829 |
| TIMP-4 | 0.939 |
| MMP-2 | 0.735 |
| MMP-3 | 0.746 |
| MMP-7 | 0.716 |
| MMP-8 | 0.049 |
| MMP-9 | 0.154 |
| **LV Cardiomyocyte stress/damage** | |
| BNP | <0.0001 |
| Pro-BNP | <0.0001 |
| **Myocardial Hypertrophy** | |
| Renin | 0.447 |
| **Inflammation/oxidative stress** | |
| Myeloperoxidase | 0.428 |
| hs-CRP | 0.740 |
| TNFR-1 | 0.528 |
| Interleukin-6 | 0.300 |
| **Atrial stress/stretch** | |
| NTpro-ANP | 0.002 |
| **Renal markers** | |
| Cystatin C | 0.643 |
| NGAL | 0.593 |
| Multiple linear regression following adjustment for the following clinical variables: age, gender, body mass index, diabetes, hypertension, lung disease, atrial fibrillation, chronic kidney disease, coronary artery disease. Note: all plasma biomarkers above were log transformed prior to analysis | |
