# Supplementary material for: Characterizing heart failure with preserved and reduced ejection fraction: An imaging and plasma biomarker approach
Source: PLoS One. 2020 Apr 29;15(4):e0232280. doi: 10.1371/journal.pone.0232280 (PMC7190371; doi:10.1371/journal.pone.0232280)
Supplement: S3 Table — (DOCX) [file pone.0232280.s003.docx]

S1 Table 1: Baseline clinical characteristics of hypertensive versus non-hypertensive controls

|  | **Hypertensive controls**  **n=22 (46%)** | **Non-hypertensive**  **controls**  **n=26 (54%)** | **p value** |
| --- | --- | --- | --- |
| Age (years) | 75±6 | 71±3 | 0.006 |
| Male (%) | 13 (59) | 11 (42) | 0.247 |
| Heart rate (b.p.m) | 67±10 | 68±11 | 0.611 |
| Systolic BP (mmHg) | 160±22 | 143±24 | 0.018 |
| Diastolic BP (mmHg) | 81±12 | 78±9 | 0.365 |
| Body mass index (kg/m2) | 26±4 | 25±2 | 0.139 |
| Asthma or COPD (%) | 2 (9) | 1 (4) | 0.454 |
| Smoking (%) | 12 (55) | 5 (19) | 0.011 |
| Hypercholesterolaemia (%) | 12 (55) | 6 (23) | 0.025 |
| Betablocker (%) | 2 (9) | 0 (0) | 0.116 |
| ACEi or ARB (%) | 10 (46) | 0 (0) |  |
| 6 minute walk distance | 360 (325-423) | 410 (378-456) | 0.007 |
| Sodium (mmol/L) | 140±2 | 141±2 | 0.104 |
| Urea (mmol/L) | 6±2 | 6±1 | 0.209 |
| Creatinine (mmol/L) | 78 (59-90) | 65 (56-78) | 0.353 |
| eGFR (ml/min/m2) | 85 (70-103) | 94 (78-98) | 0.521 |
| CKD grade |  |  | 0.062 |
| 1 | 9 (41) | 17 (65) | - |
| 2 | 11 (50) | 9 (35) | - |
| 3 | 2 (9) | 0 (0) | - |
| Haemoglobin (g/L) | 141±15 | 140±14 | 0.786 |
| Haematocrit (%) | 40±4 | 41±4 | 0.718 |
| BNP (ng/L) | 35 (23-45) | 32 (24-41) | 0.394 |
| Values are mean ± SD or n (%) or median (interquartile range). ACEi = angiotensin converting enzyme inhibitor; ARB = angiotensin II receptor blocker; BNP = B-type natriuretic peptide; CKD = chronic kidney disease; COPD = chronic obstructive pulmonary disease; eGFR = estimated glomerular filtration rate | | | |
