# Supplementary material for: Characterizing heart failure with preserved and reduced ejection fraction: An imaging and plasma biomarker approach
Source: PLoS One. 2020 Apr 29;15(4):e0232280. doi: 10.1371/journal.pone.0232280 (PMC7190371; doi:10.1371/journal.pone.0232280)
Supplement: S4 Table — (DOCX) [file pone.0232280.s004.docx]

S2 Table 2: Imaging characteristics of hypertensive versus non-hypertensive controls

|  | **Hypertensive controls**  **n=22 (46%)** | **Non-hypertensive**  **controls**  **n=26 (54%)** | **p value** |
| --- | --- | --- | --- |
| Echo | | | |
| E/E’ | 9±2 | 9±3 | 0.390 |
| LAVImax (ml/m2) | 30±7 | 29±8 | 0.882 |
| LAVImin (ml/m2) | 17±5 | 16±5 | 0.743 |
| LAEF (%) | 43±10 | 44±12 | 0.948 |
| CMR | | | |
| LV | | | |
| LVEDVI (ml/m2) | 82±15 | 80±13 | 0.766 |
| LVESVI (ml/m2) | 34±9 | 34±6 | 0.920 |
| LVEF (%) | 58±6 | 58±4 | 0.540 |
| LVEDMI (g/m2) | 48±10 | 44±9 | 0.117 |
| LV mass/LV volume | 0.59±0.09 | 0.55±0.10 | 0.124 |
| RV | | | |
| RVEDVI (ml/m2) | 83±14 | 83±17 | 0.918 |
| RVESVI (ml/m2) | 36±9 | 37±10 | 0.587 |
| RVEF (%), median (range) | 56 (53-59) | 54 (51-59) | 0.331 |
| LA | | | |
| LAVImax (ml/m2) | 33±12 | 36±12 | 0.323 |
| LAVImin (ml/m2) | 17±8 | 18±8 | 0.682 |
| LA reservoir volume indexed (ml/m2) | 16±6 | 18±5 | 0.125 |
| LA conduit volume indexed (ml/m2) | 32±9 | 28±9 | 0.182 |
| LAEF (%) | 49±12 | 52±11 | 0.324 |
| LV Tissue characterization | | | |
| ECV (%) | 25±3 | 26±3 | 0.346 |
| iECV (ml/m2) | 11.3±2.5 | 10.7±3.0 | 0.505 |
| LGE positive (%) | 5 (18) | 0 (0) | 0.023 |
| LGE positive – non-MI | 5 (18) | 0 (0) | 0.023 |
| If non-MI, size of scar as % of LV mass | 2.4 (0.6–3.6) | NA | NA |
| Values are mean ± SD or n (%). CTR = cardiothoracic ratio; ECV = extracellular volume; iECV = indexed ECV; LAEF = left atrial ejection fraction; LAVI = left atrial volume indexed to body surface area (maximal/minimal); LVEDMI = left ventricular end-diastolic mass indexed to body surface area; LVEDVI = left ventricular end-diastolic volume indexed to body surface area; MI = myocardial infarction; NA = not applicable; RVEF = right ventricular ejection fraction; RVEDVI = right ventricular end-diastolic volume indexed to body surface area | | | |
