# Supplementary material for: Characterizing heart failure with preserved and reduced ejection fraction: An imaging and plasma biomarker approach
Source: PLoS One. 2020 Apr 29;15(4):e0232280. doi: 10.1371/journal.pone.0232280 (PMC7190371; doi:10.1371/journal.pone.0232280)
Supplement: S5 Table — (DOCX) [file pone.0232280.s005.docx]

S3 Table 3: Plasma biomarker profiles of hypertensive versus non-hypertensive controls

|  | **Hypertensive controls**  **n=22 (46%)** | **Non-hypertensive**  **controls**  **n=26 (54%)** | **p value** |
| --- | --- | --- | --- |
| Interstitial fibrosis | | | |
| ST-2 (ng/ml) | 5871 (4811-6988) | 5683 (3566-6932) | 0.458 |
| Galectin-3 (ng/ml) | 4853 (4332-5646) | 5358 (4123-6318) | 0.428 |
| GDF-15 (ng/ml) | 1008 (754-1249) | 937 (574-1367) | 0.428 |
| Tenascin-C (ng/ml) | 9.9 (7.7-12.1) | 11.5 (9.7-14.4) | 0.110 |
| TIMP-1 (ng/ml) | 634 (507-833) | 730 (596-956) | 0.080 |
| TIMP-4 (ng/ml) | 1.2 (1.1-1.5) | 1.4 (1.2-1.7) | 0.128 |
| MMP-2 (ng/ml) | 64.4 (56.2-67.9) | 62.1 (56.5-68.7) | 0.669 |
| MMP-3 (ng/ml) | 5.4 (3.6-8.9) | 6.0 (3.0-7.2) | 0.606 |
| MMP-7 (ng/ml) | 0.3 (0.2-0.5) | 0.3 (0.2-0.5) | 0.970 |
| MMP-8 (ng/ml) | 0.2 (0.1-0.2) | 0.2 (0.2-0.3) | 0.194 |
| MMP-9 (ng/ml) | 20.9 (16.6-29.0) | 32.0 (15.1-64.6) | 0.094 |
| LV Cardiomyocyte stress/damage | | | |
| Elevated Troponin-I, ng/L (%) | 0 (0) | 0 (0) | NA |
| BNP (ng/L) | 35 (23-45) | 32 (24-41) | 0.394 |
| Pro-BNP (pg/ml) | 1.2 (1.1-1.5) | 1.2 (1.1-1.3) | 0.520 |
| Myocardial Hypertrophy | | | |
| Renin (pg/ml) | 144 (98-207) | 99 (38-153) | 0.092 |
| Inflammation/oxidative stress | | | |
| Myeloperoxidase (ng/ml) | 151 (129-166) | 160 (135-202) | 0.279 |
| hs-CRP (ng/ml) | 4653 (2283-15892) | 10067 (4107-21542) | 0.085 |
| TNFR-1 (ng/ml) | 3.3 (2.4-3.7) | 3.2 (2.8-3.6) | 0.642 |
| Interleukin-6 (pg/ml) | 2.9 (2.4-3.1) | 2.9 (2.6-3.3) | 0.307 |
| Atrial stress/stretch | | | |
| NTpro-ANP (pg/ml) | 3979 (3300-4401) | 4059 (3593-4505) | 0.346 |
| Renal markers | | | |
| Cystatin C (ng/ml) | 600 (536-647) | 583 (517-661) | 0.970 |
| NGAL (ng/ml) | 24.4 (21.9-33.8) | 28.0 (20.4-38.8) | 0.697 |
| Values are median (IQR) or n (%). GDF-15 = growth differentiation factor-15; hs-CRP = highly-sensitive C-reactive protein; MMP = matrix metalloproteinases; NGAL = neutrophil gelatinase-associated lipocalin; NTpro-ANP = N-terminal pro-atrial natriuretic peptide; ST2 = suppression of tumorigencity-2 ; TIMP = tissue inhibitor of metalloproteinase; TNFR-1 = tumour necrosis factor receptor-1 | | | |
