# Supplementary material for: Characterizing heart failure with preserved and reduced ejection fraction: An imaging and plasma biomarker approach
Source: PLoS One. 2020 Apr 29;15(4):e0232280. doi: 10.1371/journal.pone.0232280 (PMC7190371; doi:10.1371/journal.pone.0232280)
Supplement: S6 Table — (DOCX) [file pone.0232280.s006.docx]

S4 Table 4: Intra-observer and inter-observer assessments for CMR parameters

| **Parameter** | **Mean difference ± SD** | **ICC** | **Variability (1 – ICC)** | **Co-efficient of Variation** | **95% Limits**  **of Agreement** |
| --- | --- | --- | --- | --- | --- |
| **LV Intra-observer** | | | | | |
| LVEF (%) | 1±3 | 0.98 | 0.02 | 4.7 | -4 to 6 |
| LVEDV (ml) | -0.3±1 | 0.99 | 0.01 | 0.4 | -3 to 2 |
| LVESV (ml) | 0.5±6 | 0.99 | 0.01 | 5.9 | -11 to 12 |
| LV mass (g) | -2±5 | 0.99 | 0.01 | 3.9 | -11 to 7 |
| **LV Inter-observer** | | | | | |
| LVEF (%) | 3±4 | 0.91 | 0.09 | 8.1 | -6 to 12 |
| LVEDV (ml) | -11±12 | 0.97 | 0.03 | 6.3 | -34 to 12 |
| LVESV (ml) | -10±15 | 0.96 | 0.04 | 16.6 | -39 to 19 |
| LV mass (g) | 0.6±9 | 0.97 | 0.03 | 7.7 | -17 to 18 |
| **RV Intra-observer** | | | | | |
| RVEF (%) | 0.8±3 | 0.95 | 0.05 | 6.8 | -6 to 7 |
| RVEDV (ml) | - 2±7 | 0.99 | 0.01 | 3.5 | -16 to 12 |
| RVESV (ml) | -2±6 | 0.99 | 0.01 | 5.9 | -14 to 10 |
| **RV Inter-observer** | | | | | |
| RVEF (%) | 4±6 | 0.79 | 0.21 | 11.1 | -7 to 15 |
| RVEDV (ml) | -3±15 | 0.98 | 0.02 | 7.6 | -33 to 27 |
| RVESV (ml) | -10±17 | 0.96 | 0.04 | 16.4 | -43 to 23 |
| **LA Intra-observer** | | | | | |
| LAVmin (ml) | 1±4 | 0.99 | 0.01 | 5.4 | -7 to 8 |
| LAVmax (ml) | 2±5 | 0.99 | 0.01 | 4.8 | -7 to 12 |
| LAEF (%) | 0.1±3 | 0.98 | 0.02 | 9.4 | -6 to 6 |
| **LA Inter-observer** | | | | | |
| LAVmin (ml) | 0.7±5 | 0.99 | 0.01 | 6.8 | -9 to 10 |
| LAVmax (ml) | 3±6 | 0.99 | 0.01 | 6.3 | -10 to 15 |
| LAEF (%) | 2±4 | 0.95 | 0.05 | 12.2 | -6 to 10 |
| **Fibrosis Intra-observer** | | | | | |
| Total focal fibrosis (g) | 0±0.2 | 0.99 | 0.01 | 6.6 | -0.4 to 0.4 |
| ECV (%) | -0.5±1.3 | 0.96 | 0.04 | 4.6 | -2.9 to 2.1 |
| Myocardial Native T1 (ms) | 7±13.7 | 0.99 | 0.01 | 1.1 | -19.8 to 33.8 |
| Post-contrast T1 (ms) | -1.9±2.8 | 0.99 | 0.01 | 0.6 | -7.4 to 3.5 |
| **Fibrosis Inter-observer** | | | | | |
| Total focal fibrosis (g) | - 0.1±0.3 | 0.99 | 0.01 | 8.6 | - 0.4 to 0.7 |
| ECV (%) | -0.3±1.3 | 0.96 | 0.04 | 4.6 | -2.8 to 2.2 |
| Myocardial Native T1 (ms) | 16.4±31 | 0.95 | 0.05 | 2.5 | -44.4 to 77.1 |
| Post-contrast T1 (ms) | -0.5±3.6 | 0.99 | 0.01 | 0.8 | -7.5 to 6.5 |
| Abbreviations are as for main Table 2; ICC = intra-class correlation coefficient | | | | | |
