# Supplementary material for: Characterizing heart failure with preserved and reduced ejection fraction: An imaging and plasma biomarker approach
Source: PLoS One. 2020 Apr 29;15(4):e0232280. doi: 10.1371/journal.pone.0232280 (PMC7190371; doi:10.1371/journal.pone.0232280)
Supplement: S7 Table — (DOCX) [file pone.0232280.s007.docx]

S5 Table 5: Significant associations of the presence of focal fibrosis on late gadolinium enhancement imaging with other plasma biomarkers

|  | **Correlation coefficients (Spearman’s)** | **P value** |
| --- | --- | --- |
| **Plasma markers of interstitial fibrosis** | | |
| Galectin3 | 0.149 | 0.029 |
| GDF-15 | 0.235 | 0.001 |
| MMP-3 | 0.188 | 0.006 |
| MMP-7 | 0.270 | <0.0001 |
| MMP-8 | 0.159 | 0.021 |
| **Plasma markers of cardiomyocyte stress/damage** | | |
| BNP | 0.371 | 0.0001 |
| pro-BNP | 0.203 | 0.003 |
| NTpro-ANP | 0.209 | 0.002 |
