# Supplementary material for: Characterizing heart failure with preserved and reduced ejection fraction: An imaging and plasma biomarker approach
Source: PLoS One. 2020 Apr 29;15(4):e0232280. doi: 10.1371/journal.pone.0232280 (PMC7190371; doi:10.1371/journal.pone.0232280)
Supplement: S8 Table — (DOCX) [file pone.0232280.s008.docx]

S6 Table 6: Significant associations of diffuse fibrosis (extracellular volume) on CMR with other plasma biomarkers

|  | **Correlation coefficients (Spearman’s)** | **P value** |
| --- | --- | --- |
| **Plasma markers of interstitial fibrosis** | | |
| GDF15 | 0.210 | 0.007 |
| Tenascin-C | 0.156 | 0.046 |
| MMP-2 | 0.218 | 0.005 |
| MMP-3 | 0.172 | 0.027 |
| MMP-7 | 0.183 | 0.019 |
| **Plasma markers of cardiomyocyte stress/damage** | | |
| BNP | 0.408 | <0.0001 |
| proBNP | 0.377 | <0.0001 |
| NTproANP | 0.369 | <0.0001 |
