# Supplementary material for: Characterizing heart failure with preserved and reduced ejection fraction: An imaging and plasma biomarker approach
Source: PLoS One. 2020 Apr 29;15(4):e0232280. doi: 10.1371/journal.pone.0232280 (PMC7190371; doi:10.1371/journal.pone.0232280)
Supplement: S9 Table — (DOCX) [file pone.0232280.s009.docx]

S7 Table 7: Baseline clinical characteristics of heart failure sub-groups following exclusion of known coronary artery disease and/or MI on LGE

|  | **HFpEF**  **n=97** | **HFrEF**  **n=18** | **p value** |
| --- | --- | --- | --- |
| Age (years) | 72±10 | 70±11 | 0.370 |
| Male (%) | 44 (45) | 8 (44) | 0.943 |
| Heart rate (b.p.m) | 71±14 | 69±20 | 0.577 |
| Systolic BP (mmHg) | 143±26 | 136±22 | 0.310 |
| Diastolic BP (mmHg) | 74±13 | 70±22 | 0.276 |
| Body mass index (kg/m2) | 34±8 | 31±7 | 0.077 |
| Sinus Rhythm (%) | 70 (72) | 13 (72) | 0.996 |
| Atrial Fibrillation | 27 (28) | 5 (28) | 0.996 |
| Diabetes (%) | 45 (46) | 9 (50) | 0.778 |
| Hypertension (%) | 86 (89) | 10 (56) | 0.001 |
| Asthma or COPD (%) | 17 (18) | 1 (6) | 0.199 |
| Smoking (%) | 47 (49) | 6 (33) | 0.237 |
| Hypercholesterolaemia (%) | 48 (50) | 5 (28) | 0.090 |
| Peripheral Vascular Disease (%) | 1 (1) | 0 (0) | 0.665 |
| TIA or CVA (%) | 13 (14) | 3 (17) | 0.726 |
| Betablocker (%) | 64 (66) | 14 (78) | 0.325 |
| ACEi or ARB (%) | 83 (86) | 16 (89) | 0.708 |
| Aldosterone antagonist (%) | 29 (30) | 5 (28) | 0.856 |
| Loop Diuretic (%) | 79 (81) | 15 (83) | 0.849 |
| NYHA III/IV (%) | 31 (32) | 4 (22) | 0.410 |
| 6 minute walk distance | 180 (120-250) | 230 (130-250) | 0.081 |
| MLWHF score | 50 (25-66) | 49 (30-71 | 0.946 |
| Sodium (mmol/L) | 139±4 | 140±2 | 0.090 |
| Urea (mmol/L) | 8±4 | 10±5 | 0.181 |
| Creatinine (mmol/L) | 88 (72-113) | 98 (75-135) | 0.455 |
| eGFR (ml/min/m2) | 68 (53-84) | 61 (45-79) | 0.409 |
| CKD grade |  |  | 0.714 |
| 1 | 22 (23) | 3 (17) |  |
| 2 | 37 (38) | 6 (33) |  |
| 3 | 38 (39) | 9 (50) |  |
| Haemoglobin (g/L) | 127±23 | 131±17 | 0.539 |
| Haematocrit (%) | 38±7 | 39±5 | 0.539 |
| BNP (ng/L) | 133 (53-239) | 464 (161-769) | <0.0001 |
| Values are mean ± SD or n (%) or median (interquartile range). ACEi = angiotensin converting enzyme inhibitor; ARB = angiotensin II receptor blocker; BNP = B-type natriuretic peptide; CKD = chronic kidney disease; COPD = chronic obstructive pulmonary disease; eGFR = estimated glomerular filtration rate | | | |
