# Supplementary material for: Characterizing heart failure with preserved and reduced ejection fraction: An imaging and plasma biomarker approach
Source: PLoS One. 2020 Apr 29;15(4):e0232280. doi: 10.1371/journal.pone.0232280 (PMC7190371; doi:10.1371/journal.pone.0232280)
Supplement: S10 Table — (DOCX) [file pone.0232280.s010.docx]

S8 Table 8: Imaging characteristics of heart failure sub-groups following exclusion of known coronary artery disease and/or MI on LGE

|  | **HFpEF**  **n=97** | **HFrEF**  **n=18** | **p value** |
| --- | --- | --- | --- |
| **Prior Chest Radiography** | | | |
| Pulmonary oedema (%) | 66 (69) | 12 (71) | 0.880 |
| Raised CTR (%) | 65 (68) | 14 (82) | 0.225 |
| Pleural effusion (%) | 33 (34) | 9 (53) | 0.144 |
| **Echo** | | | |
| E/E’ | 13±5 | 16±6 | 0.025 |
| LAVImax (ml/m2) | 42±18 | 50±14 | 0.079 |
| LAVImin (ml/m2) | 29±19 | 37±14 | 0.079 |
| LAEF (%) | 36±18 | 27±12 | 0.013 |
| **CMR** | | | |
| **LV** | | | |
| LVEDVI (ml/m2) | 78±18 | 141±42 | <0.0001 |
| LVESVI (ml/m2) | 34±10 | 103±44 | <0.0001 |
| LVEF (%) | 57±6 | 29±9 | <0.0001 |
| LVEDMI (g/m2) | 51±14 | 68±25 | <0.0001 |
| LV mass/LV volume | 0.66±0.15 | 0.50±0.19 | <0.0001 |
| **RV** | | | |
| RVEDVI (ml/m2) | 80±20 | 92±26 | 0.030 |
| RVESVI (ml/m2) | 37±14 | 54±26 | 0.014 |
| RVEF (%), median (range) | 55 (27-69) | 44 (21-61) | <0.0001 |
| RV Dysfunction (%) | 16 (17) | 10 (56) | <0.0001 |
| **LA – sinus rhythm subjects** | | | |
| LAVImax (ml/m2) | 43±17 | 61±21 | 0.001 |
| LAVImin (ml/m2) | 26±13 | 44±20 | <0.0001 |
| LA reservoir volume indexed (ml/m2) | 17±6 | 19±6 | 0.228 |
| LA conduit volume indexed (ml/m2) | 28±8 | 18±8 | <0.0001 |
| LAEF (%) | 41±12 | 33±11 | 0.030 |
| **LV Tissue characterization** | | | |
| ECV (%) | 27±5 | 27±3 | 0.662 |
| iECV (ml/m2) | 13.0±4.0 | 16.6±6.1 | 0.006 |
| LGE positive – non-MI | 36 (38) | 13 (72) | 0.006 |
| Values are mean ± SD or n (%). CTR = cardiothoracic ratio; ECV = extracellular volume; iECV = indexed ECV; LAEF = left atrial ejection fraction; LAVI = left atrial volume indexed to body surface area (maximal/minimal); LVEDMI = left ventricular end-diastolic mass indexed to body surface area; LVEDVI = left ventricular end-diastolic volume indexed to body surface area; MI = myocardial infarction; NA = not applicable; RVEF = right ventricular ejection fraction; RVEDVI = right ventricular end-diastolic volume indexed to body surface area | | | |
