# Supplementary material for: Characterizing heart failure with preserved and reduced ejection fraction: An imaging and plasma biomarker approach
Source: PLoS One. 2020 Apr 29;15(4):e0232280. doi: 10.1371/journal.pone.0232280 (PMC7190371; doi:10.1371/journal.pone.0232280)
Supplement: S11 Table — (DOCX) [file pone.0232280.s011.docx]

S9 Table 9: Plasma biomarker profiles of heart failure sub-groups following exclusion of known coronary artery disease and/or MI on LGE

|  | **HFpEF**  **n=97** | **HFrEF**  **n=18** | **p value** |
| --- | --- | --- | --- |
| **Interstitial fibrosis** | | | |
| ST-2 (ng/ml) | 6386 (4956-8915) | 5117 (3833-7708) | 0.082 |
| Galectin-3 (ng/ml) | 7387 (5509-8878) | 7431 (6549-10348) | 0.252 |
| GDF-15 (ng/ml) | 2180 (1546-3562) | 2133 (1562-4531) | 0.655 |
| Tenascin-C (ng/ml) | 13.6 (10.9-17.4) | 13.1 (11.1-20.6) | 0.935 |
| TIMP-1 (ng/ml) | 982 (693-1420) | 1115 (858-1329) | 0.423 |
| TIMP-4 (ng/ml) | 1.7 (1.4-2.2) | 1.8 (1.4-2.2) | 1.000 |
| MMP-2 (ng/ml) | 72.5 (58.6-86.0) | 76.7 (66.3-88.5) | 0.204 |
| MMP-3 (ng/ml) | 6.6 (4.5-10.4) | 8.3 (4.5-13.3) | 0.423 |
| MMP-7 (ng/ml) | 0.6 (0.4-1.0) | 0.8 (0.5-1.6) | 0.218 |
| MMP-8 (ng/ml) | 0.3 (0.2-0.5) | 0.3 (0.2-0.6) | 0.633 |
| MMP-9 (ng/ml) | 30.7 (19.3-58.9) | 36.6 (19.6-58.1) | 0.720 |
| **LV Cardiomyocyte stress/damage** | | | |
| Elevated Troponin-I, ng/L (%) | 19 (20) | 9 (50) | 0.006 |
| BNP (ng/L) | 133 (53-239) | 464 (161-769) | <0.0001 |
| Pro-BNP (pg/ml) | 1.6 (1.2-2.2) | 2.6 (1.6-5.9) | 0.001 |
| **Myocardial Hypertrophy** | | | |
| Renin (pg/ml) | 352 (186-824) | 309 (209-1266) | 0.509 |
| **Inflammation/oxidative stress** | | | |
| Myeloperoxidase (ng/ml) | 211 (159-262) | 258 (199-315) | 0.079 |
| hs-CRP (ng/ml) | 47869 (14421-87454) | 24604 (9012-77860) | 0.331 |
| TNFR-1 (ng/ml) | 5.4 (4.0-8.1) | 6.0 (4.2-8.1) | 0.633 |
| Interleukin-6 (pg/ml) | 3.9 (3.2-4.8) | 3.7 (3.0-4.5) | 0.408 |
| **Atrial stress/stretch** | | | |
| NTpro-ANP (pg/ml) | 6439 (3874-8304) | 8017 (6382-10492) | 0.012 |
| **Renal markers** | | | |
| Cystatin C (ng/ml) | 757 (658-1016) | 908 (713-1038) | 0.230 |
| NGAL (ng/ml) | 46.3 (32.5-63.7) | 47.4 (36.3-74.0) | 0.418 |
| Values are median (IQR) or n (%). GDF-15 = growth differentiation factor-15; hs-CRP = highly-sensitive C-reactive protein; MMP = matrix metalloproteinases; NGAL = neutrophil gelatinase-associated lipocalin; NTpro-ANP = N-terminal pro-atrial natriuretic peptide; ST2 = suppression of tumorigencity-2 ; TIMP = tissue inhibitor of metalloproteinase; TNFR-1 = tumour necrosis factor receptor-1 | | | |
