# Supplementary figures and images for: Characterizing heart failure with preserved and reduced ejection fraction: An imaging and plasma biomarker approach
Source: PLoS One. 2020 Apr 29;15(4):e0232280. doi: 10.1371/journal.pone.0232280 (PMC7190371; doi:10.1371/journal.pone.0232280)

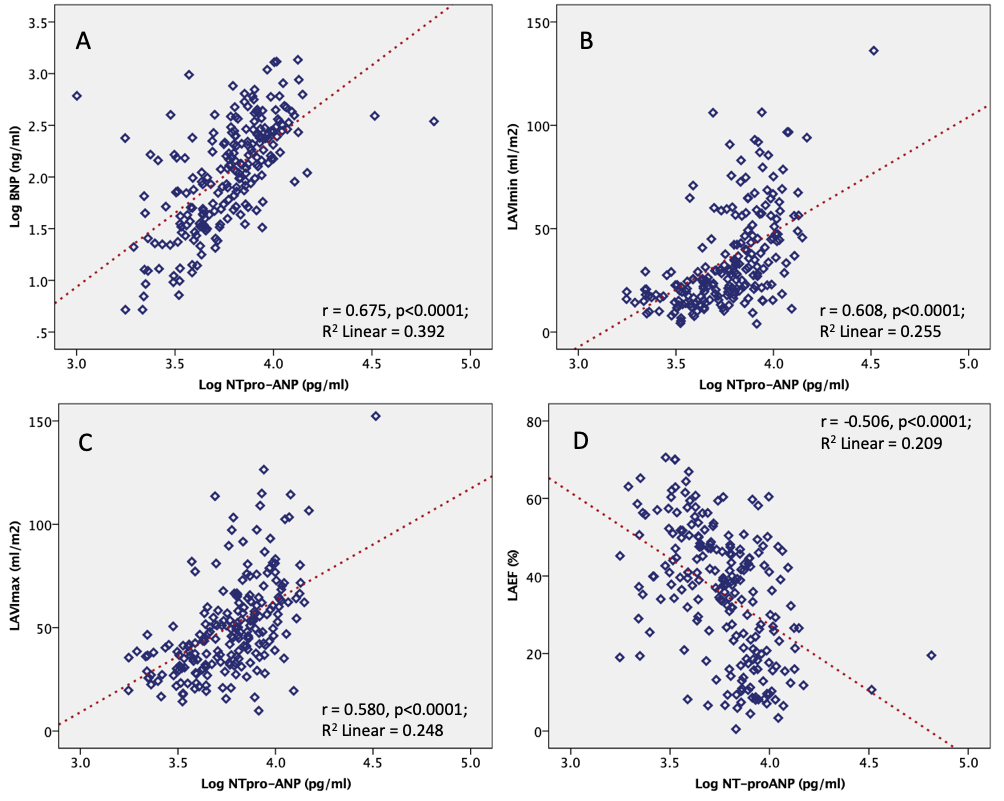

Supplement: S1 Fig — Scatter plots illustrating the relationship between NTpro-ANP and: A) BNP B) minimum left atrium volume indexed–LAVImin C) maximum left atrium volume indexed—LAVImax D) left atrial ejection fraction. (TIFF) [file pone.0232280.s012.tiff]

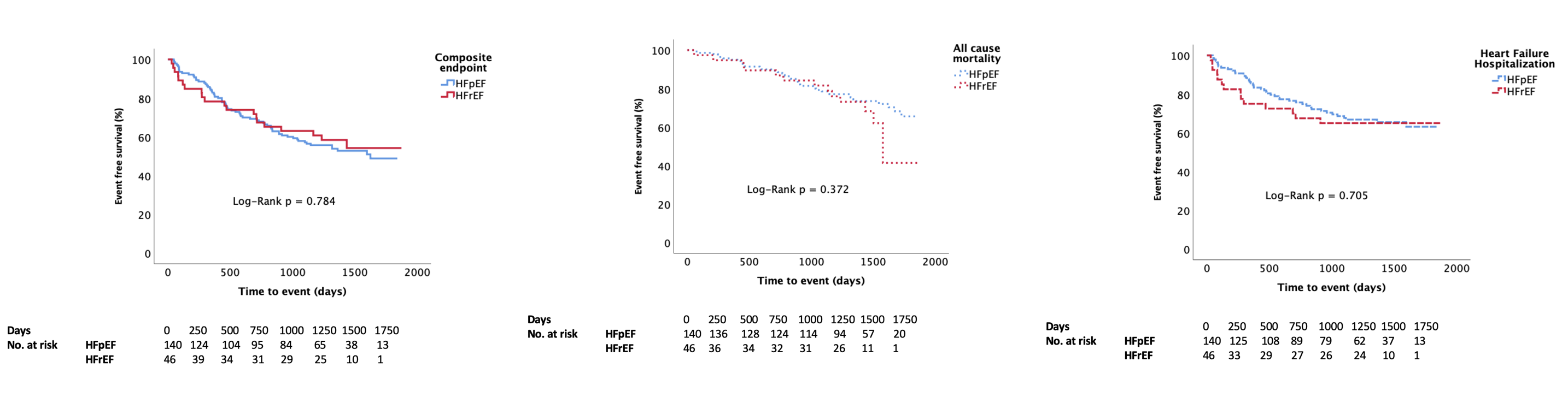

Supplement: S2 Fig — Survival curves stratified according to heart failure groups for the primary endpoint: composite of all-cause mortality or hospitalization for HF (left panel); all-cause mortality (middle panel); HF hospitalization (right panel). (TIFF) [file pone.0232280.s013.tiff]
